# Feasibility, safety, and resource utilisation of active mobilisation of patients on extracorporeal life support: a prospective observational study

**DOI:** 10.1186/s13613-020-00776-3

**Published:** 2020-12-01

**Authors:** Stephan Braune, Patrick Bojes, Anne Mecklenburg, Federico Angriman, Gerold Soeffker, Katja Warnke, Dirk Westermann, Stefan Blankenberg, Mathias Kubik, Hermann Reichenspurner, Stefan Kluge

**Affiliations:** 1 Department of Intensive Care Medicine, University Medical Center Hamburg-Eppendorf, Martinistr. 52, 20246 Hamburg, Germany; 2 Interdepartmental Division of Critical Care Medicine, Sunnybrook Health Sciences Centre, Toronto, Canada; 3 Department of Interventional and General Cardiology, University Heart & Vascular Center Hamburg, Hamburg, Germany; 4 Department of Cardiovascular Surgery, University Heart & Vascular Center Hamburg, Hamburg, Germany

**Keywords:** Mobilisation, Extracorporeal life support, ECLS, ECMO, ECCO_2_R

## Abstract

**Background:**

There is scarce evidence on the feasibility, safety and resource utilisation of active mobilisation in critically ill patients on extracorporeal life support (ECLS).

**Methods:**

This prospective observational single-centre study included all consecutive critically ill patients on ECLS admitted to an academic centre in Germany over a time period of one year. The level of mobilisation was categorised according to the ICU Mobility Scale (IMS). Primary outcome was complications during mobilisation.

**Results:**

During the study period, active mobilisation with an activity level on the IMS of ≥ 3 was performed at least on one occasion in 43 out of 115 patients (37.4%). A total of 332 mobilisations with IMS ≥ 3 were performed during 1242 ECLS days (26.7%). ECLS configurations applied were va-ECMO (*n* = 63), vv-ECMO (*n* = 26), vv-ECCO_2_R (*n* = 12), av-ECCO_2_R (*n* = 10), and RVAD (*n* = 4). Femoral cannulation had been in place in 108 patients (93.9%). The median duration of all mobilisation activities with IMS ≥ 3 was 130 min (IQR 44–215). All mobilisations were undertaken by a multi-professional ECLS team with a median number of 3 team members involved (IQR 3–4). Bleeding from cannulation site requiring transfusion and/or surgery occurred in 6.9% of actively mobilised patients and in 15.3% of non-mobilised patients. During one mobilisation episode, accidental femoral cannula displacement occurred with immediate and effective recannulation. Sedation was the major reason for non-mobilisation.

**Conclusions:**

Active mobilisation (IMS ≥ 3) of ECLS patients undertaken by an experienced multi-professional team was feasible, and complications were infrequent and managed successfully. Larger prospective multicentre studies are needed to further evaluate early goal directed sedation and mobilisation bundles in patients on ECLS.

## Background

Prolonged immobilisation during critical illness often promotes neuromuscular and neuropsychiatric syndromes such as intensive care unit-acquired weakness (ICU-AW) and ICU delirium, which in turn is associated with relevant long-term post-ICU morbidity [1]. Clinical Practice Guidelines for the Management of Pain, Agitation, and Delirium have been published in order to enhance liberation from mechanical ventilation, target adequate sedation, increase patients’ functional status, and reduce neuromuscular and neuropsychiatric side-effects [2, 3]. Early and active mobilisation is associated with higher chances of returning to independent functioning, lower incidence of delirium, as well as decreased duration of mechanical ventilation, ICU and hospital length of stay [4]. Despite growing evidence that active mobilisation of critically ill patients is not only beneficial, but also safe and feasible in the general ICU population, many barriers to its implementation in routine clinical practice have been identified [5–8].

Over the last decade, extracorporeal life support (ECLS) is increasingly being applied to critically ill patients with severe circulatory and/or respiratory failure [9, 10]. Technological advances improving the quality and safety of membranes, circuits, cannulas, and pumps as well as promising clinical research results on the potential benefit of ECLS on survival have all motivated this trend [9]. However, patients receiving ECLS are often considered too unstable for active mobilisation [11, 12]. Moreover, concerns about specific complications associated with active mobilisation of patients on ECLS, such as circuit malfunction and/or cannula complications, are potential barriers to active mobilisation of these patients [11, 12]. So far, only a few retrospective cohort studies and one recent small pilot randomised controlled study have been published on the feasibility and safety of actively mobilising critically ill patients on ECLS [13, 14].

This prospective observational single-centre study was initiated within a quality assurance programme to evaluate the feasibility and safety of active mobilisation in ECLS patients by a multi-professional team. Further goals were to assess resource requirements for active mobilisations, as well as to investigate reasons for withholding active mobilisation to patients on ECLS.

## Methods

### Study design and population

This prospective observational study was conducted in a university hospital and ECLS referral centre in Germany. All consecutive adult patients admitted to the department of intensive care medicine from January to December 2014 treated with percutaneous ECLS for severe circulatory and/or respiratory failure were included. The study protocol was approved by the institutional ethics committee (protocol number PV4685) and was conducted according to the amended declaration of Helsinki.

ECLS configurations under investigation were veno-arterial extracorporeal membrane oxygenation (va-ECMO), veno-venous extracorporeal membrane oxygenation (vv-ECMO), veno-venous extracorporeal carbon dioxide removal (vv-ECCO_2_R), arterio-venous extracorporeal carbon dioxide removal (av-ECCO_2_R), and right ventricular assist device (RVAD).

### Clinical setting and protocol

The multi-professional ECLS team comprised critical care nurses, physiotherapists, perfusionists, intensive care physicians, and cardiac surgeons. Local protocols for ECLS management, anticoagulation, mechanical ventilation, analgosedation, weaning and mobilisation were followed. All ECLS cannulas were secured by means of cutaneous sutures in combination with adhesive tapes and bandages. In va-ECMO a distal perfusion catheter was placed in addition to the arterial cannula to enhance distal limb perfusion.

All ECLS patients were screened daily for readiness for mobilisation. The standardised screening procedure included assessment of neurological ability, cardiopulmonary stability, stability of the ECLS circuit flow, and securement of the ECLS cannulas. In patients with femoral cannulation a 90° hip flexion was performed to ensure stable ECLS blood flow under flexed conditions. Thereafter, a daily individualised mobilisation plan was implemented by the interprofessional team, led by the physiotherapists, mobilising ECLS patients as far as clinically feasible and appropriate. The mobilisation sessions included functional strengthening, breathing exercises, active upper and lower limb exercises, endurance exercises, and progressing functional mobility. The level of mobilisation on each day on ECLS was categorised according to the ICU Mobility Scale (IMS; Table 1) [15].

**Table 1:** ICU mobility scale [15]

ICU mobility scale (IMS)
IMS 0: no mobilisation or passively exercised by staff
IMS 1: sitting in bed and actively exercising
IMS 2: passively moved to chair without standing
IMS 3: sitting over edge of bed
IMS 4: standing in front of bed
IMS 5: transferring bed to chair
IMS 6: marching on spot
IMS 7: walking with assistance of more than one person
IMS 8: walking with assistance of one person
IMS 9: walking independently with a gait aid
IMS 10: walking independently without a gait aid

*ICU* intensive care unit, *IMS* ICU mobility scale

Reasons for deferring active mobilisation per protocol included severe hypoxemia, hemodynamic instability, unstable dysrhythmia, bleeding or high risk of bleeding, sedation or coma precluding active mobilisation and the use of neuromuscular blockade.

To manage equipment and continuously monitor haemodynamic and respiratory state, the multi-professional team mobilising the patient typically consisted of a physiotherapist, a critical care nurse, and an intensivist. Prior to each intervention in patients with femoral cannulas the team performed a hip flexion manoeuvre to ascertain stable extracorporeal blood flow and secure cannulas. Non-essential treatments were temporarily discontinued during active mobilisation. Based on clinical judgement extracorporeal respiratory and/or circulatory support was temporarily increased during active mobilisation. Active mobilisation was interrupted or terminated for unexpected changes including sustained haemodynamic instability, hypoxaemia, weakness, chest pain or other relevant clinical symptoms or signs.

### Data collection

Baseline demographic and clinical data including ECLS characteristics and functions, as well as ICU-mortality was collected in the departments’ electronic patient data management system (Integrated Care Manager®, Dräger, Lübeck, Germany). As part of a one-year quality assurance project, the following data were prospectively collected before, during and after all active mobilisations at an activity level of equal to or more than side-of-bed activities (IMS ≥ 3): ECLS and cannulation settings, mechanical ventilation, concurrent renal replacement therapy, haemodynamic and respiratory status, level of sedation according to the Richmond Agitation Sedation Scale (RASS), and complications potentially related to mobilisation on ECLS.

Furthermore, the time from start to end of mobilisation and the number of team members involved in each mobilisation activity at an activity level of an IMS ≥ 3 were observed. Additionally, daily reasons for non-mobilisation as indicated by the attending ECLS team were prospectively recorded through a questionnaire.

Outcomes recorded were all ECLS-associated complications that occurred during active mobilisation as well as length of ECLS treatment, length of ICU and hospital stay, and ICU-mortality. Complications included patient-related complications (fall, haemodynamic instability, desaturation to < 85%, cardiac arrest, arrhythmia, acute limb ischemia, bleeding at cannulation site, cannula dislodgement) and circuit-related complications (oxygenator and pump failure, tubing rupture, critical drop or interruption of extracorporeal blood flow, clotting in the extracorporeal circuit). Major complications were defined as adverse events requiring abrupt discontinuation of mobilisation for medical and/or surgical treatment. Minor complications were defined as self-limiting or instantly reversible adverse events not requiring discontinuation of mobilisation.

### Statistical analyses

Continuous variables are expressed as medians (with range). Categorical variables are expressed as counts and percentages. In order to compare outcome variables between subgroups, non-parametric analyses were performed. Depending on the number of groups compared and the type of data, Chi-squared test, Fisher’s exact test, Mann–Whitney U test, or Kruskal–Wallis one-way analysis of variance were applied. A two-sided p < 0.05 was considered significant. The software used for analyses was SPSS (version 23, IBM SPSS Statistics, USA) and STATA v.14.2 (StataCorp LLC, College Station, TX, USA).

## Results

### Baseline clinical characteristics

Details of demographic baseline data, diagnostic categories and clinical severity scores differentiating for mobilised (*Mob*, IMS ≥ 3) and non-mobilised (*Non-Mob*, IMS < 3) patients are displayed in Table 2. During the study period, a total of 115 patients (32.2% female) with a median age of 57.0 years (IQR 46.0–67.5) were treated with ECLS. Mobilisation at an activity level of IMS ≥ 3 was performed at least on one occasion in 43 patients (37.4%). These patients had significant lesser severity of illness scores than those patients not mobilised to that activity level.

**Table 2:** Baseline demographics, diagnostic categories, severity of illness scores, type of primary ECLS, non-intubated patients, cannulation configuration according to level of mobilisation

Variable	Mob	Non-Mob	All	*p* value
	*n* = 43 (37.4%)	*n* = 72 (62.6%)	*n* = 115	
Age, BMI, and sex—median (IQR), *n* (%)				
Age (years)	53.0 (45.0–68.0)	58.5 (47.8–67.0)	57.0 (46.0–67.5)	0.51
Body mass index (BMI)	24.6 (21.2–27.3)	26.2 (23.2–29.3)	25.6 (22.5–27.8)	0.37
Female sex (% within subgroup)	15 (34.9)	22 (30.6)	37 (32.2)	0.68
Primary diagnosis—*n* (% Mob/Non-Mob per subgroup; % between diagnostic groups for ALL)				
ARDS	13 (34.2)	25 (65.8)	38 (33,0)	0.08
AHF without CS	10 (37.0)	17 (63.0)	27 (23,5)	0.96
AHF peri/post-CS	6 (35.3)	11 (64.7)	17 (14,8)	0.84
eCPR	3 (17.6)	14 (82.4)	17 (14,8)	0.10
COPD	7 (63.6)	4 (36.4)	11 (9,6)	0.09
ILD	3 (75.0)	1 (25.0)	4 (3,5)	0.14
PAH	1 (100.0)	0 (0.0)	1 (0,9)	0.37
ECLS as bridge-to-transplant	2 (100.0)	0 (0.0)	2 (1.7)	0.14
Severity of illness scores—median (IQR)				
SAPS-II score on ICU admission	38 (31–47)	44 (39–54)	42 (36–50)	0.01
APACHE II score on ICU admission	19 (16–22)	26 (20–30)	23 (18–28)	< 0.01
SOFA score on ICU admission	8 (6–11)	11 (8–13)	10 (7–13)	< 0.01
Type of primary ECLS—*n* (% Mob/Non-Mob per subgroup; % within ECLS types for ALL)				
va-ECMO (non-eCPR)	14 (29.8)	33 (70.2)	47 (40.9)	< 0.01
eCPR (va-ECMO)	3 (17.6)	14 (82.4)	17 (14.8)	0.01
vv-ECMO	12 (46.2)	14 (53.8)	26 (22.6)	0.84
vv-ECCO_2_R	7 (58.3)	5 (41.7)	12 (10.4)	0.77
av-ECCO_2_R	3 (33.3)	6 (66.7)	9 (7.8)	0.75
RVAD	4 (100)	0 (0)	4 (2.6)	0.02
Non-intubated ECLS—*n* (% Mob/Non-Mob per subgroup; % within ECLS types for ALL)				
All non-intubated patients on ECLS	9 (75.0)	3 (25.0)	12 (10.4)	0.14
Cannulation—*n* (% Mob/Non-Mob per subgroup; % within cannulation categories for ALL)				
Femoro-femoral	22 (31.0)	49 (69.0)	71 (61.7)	< 0.01
DL femoral	2 (100)	0 (0)	2 (1.7)	0.14
Femoro-jugular	12 (46.2)	14 (53.8)	26 (22.6)	0.85
DL jugular	3 (75.0)	1 (25.0)	4 (3.5)	0.63
Femoro-central	4 (44.4)	5 (55.6)	9 (7.8)	0.98
Central	0 (0)	3 (100)	3 (2.6)	0.29

Data are presented as *n* (%) for categorical variables and median (25th and 75th percentile) for continuous variables

*Mob* mobilised with IMS ≥ 3, *Non-Mob* never mobilised with IMS ≥ 3, *ARDS* acute respiratory distress syndrome, *COPD* chronic obstructive pulmonary disease, *ILD* interstitial lung disease, *PAH* pulmonary arterial hypertension, *AHF* acute heart failure, *CS *cardiac surgery, *eCPR* extracorporeal cardiopulmonary resuscitation, *ECLS* extracorporeal life support, *ICU* intensive care unit, *SAPS* Simplified Acute Physiology Score, *APACHE* Acute Physiology and Chronic Health Evaluation, *SOFA* Sepsis-related Organ Failure Assessment, *eCPR* extracorporeal cardiopulmonary resuscitation, *ECLS* extracorporeal life support, *va-ECMO* veno-arterial extracorporeal membrane oxygenation, *vv-ECMO* veno-venous extracorporeal membrane oxygenation, *vv-ECCO2R* veno-venous extracorporeal carbon dioxide removal, *av-ECCO2R* arterio-venous extracorporeal carbon dioxide removal, *RVAD* right ventricular assist device, *DL* double lumen

Study patients were treated with the following types of ECLS: va-ECMO (*n* = 63), vv-ECMO (*n* = 26), vv-ECCO_2_R (*n* = 12), av-ECCO_2_R (*n* = 10), and RVAD (*n* = 4). In 12 patients (10.4%) ECLS was performed in spontaneously breathing patients without invasive mechanical ventilation and in 17 patients (14.8%) more than one type of ECLS was used during their clinical course. Femoral cannulation was in place in 108 patients (93.9%). Details of respiratory, haemodynamic, and renal variables before initiation of ECLS are presented in Additional file 1

### Mobilisations

Details on how many mobilisation units were applied for each type of ECLS and cannulation configuration are given in Table 3. On 310 out of 1242 ECLS days (24.9%) a total of 332 mobilisations with an IMS ≥ 3 were undertaken. On all other ECLS days the level of mobilisation was below IMS 3. Of all 332 mobilisation units, 313 (94.3%) mobilisations were undertaken with femoral ECLS cannulation and 91 (27.4%) mobilisations with concurrent renal replacement therapy through an additional Shaldon catheter. A total of 239 (72.0%) mobilisations were performed on spontaneously breathing patients without an artificial airway, 71 (21.4%) mobilisations on patients ventilated through a tracheal tube, and 22 (6.6%) mobilisations on patients with orotracheal intubation.

**Table 3:** Active mobilisation units (IMS ≥ 3) according to ECLS type and cannulation

Variable	Mobilisation units
	*n* = 332
ECLS type—*n* (%)	
va-ECMO	72 (21.7)
vv-ECMO	100 (30.1)
vv-ECCO_2_R	48 (14.5)
av-ECCO_2_R	63 (19.0)
RVAD	49 (14.7)
Cannulation configuration—*n* (%)	
DL jugular (vv-ECCO_2_R)	19 (5.7)
Femoral any cannulation	313 (94.3)
Femoro-femoral	154 (46.4)
Femoro-femoral (va-ECMO)	72 (21.7)
Femoro-femoral (av- ECCO_2_R)	63 (19.0)
Femoro-femoral (vv-ECMO)	19 (5.7)
DL femoral (vv-ECCO_2_R)	26 (7.8)
Femoro-jugular	84 (25.3)
Femoro-jugular (vv-ECMO)	81 (24.4)
Femoro-jugular (vv-ECCO_2_R)	3 (0.9)
Femoro-central	49 (14.8)
Femoro-central (va-ECMO)	8 (2.4)
Femoro-central (RVAD)	41 (12.4)

Data are presented as *n* (%) for categorical variables. *ECLS* extracorporeal life support, *va-ECMO* veno-arterial extracorporeal membrane oxygenation, *vv-ECMO* veno-venous extracorporeal membrane oxygenation, *vv-ECCO*_*2*_*R* veno-venous extracorporeal carbon dioxide removal, *av-ECCO*_*2*_*R* arterio-venous extracorporeal carbon dioxide removal, *RVAD* right ventricular assist device, *DL* double lumen

### Duration of mobilisation and team resources

**Fig. 1:**
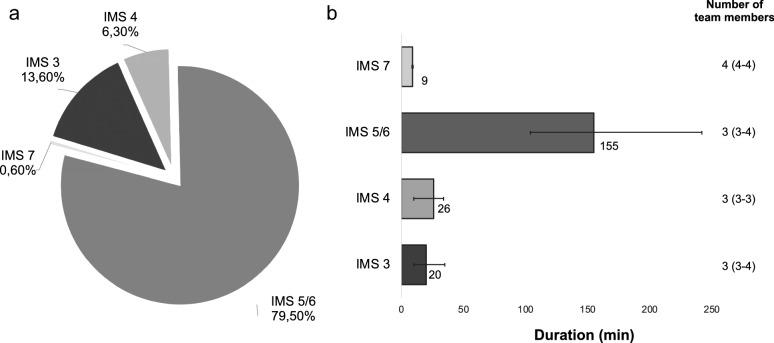
Percentage, duration and team resources according to different levels of active mobilisation (IMS 3–7). Detailed analysis of **a** the percentage of each level of mobilisation of all mobilisation units (*n* = 332), and **b** duration of different levels of mobilisation and the respective number of team members used for mobilisation. *IMS* ICU mobility scale, data presented as percent (%) and median (IQR)

Duration and team resources of active mobilisations IMS 3–7 are presented in Fig. 1. The median duration of all mobilisation activities IMS ≥ 3 was 130 min (IQR 44–215) including the duration of sitting in a chair (IMS 5). Of these mobilisation episodes, 45 were side-of-bed activities (13.6%), 21 standing activities (6.3%), 264 mobilisations into the chair via standing and brief walking (79.5%), and 2 walking exercises (0.6%). All mobilisations were undertaken by a multi-professional ECLS team with a median number of 3 team members involved (IQR 3–4).

### Reasons for non-mobilisation

The reasons for non-mobilisation on 932 ECLS days were as follows: sedation with RASS ≤ − 2 (65.0%), “poor general condition” (21.1%), coma without sedation (4.6%), lack of manpower (4.0%), haemodynamic instability (3.4%), respiratory instability (3.4%), agitation with RASS ≥ 2 (2.7%), and fear of cannula complications (2.6%). In 5.9% of non-mobilisation days no specific reason was documented.

### Complications

#### Circuit malfunction and cardiopulmonary instability

Critical blood flows of < 2 L/min in high-flow ECLS and of < 0.5 L/min in low-flow ECLS, all lasting below 60 s, occurred in 3 (0.9%) and 1 (0.3%) mobilisation episodes, respectively. Acute low-flow alarms of the ECLS console were recorded during 12 mobilisation episodes (3.4%). Median variation of ECLS blood flow from before to during active mobilisation for high-flow ECLS (va-ECMO, vv-ECMO, RVAD; *n* = 224) was − 0.1 L/min (range − 3.8–1.9 L/min). For low-flow ECLS (vv-/av-ECCO_2_R; *n* = 111) the median variation of blood flow was − 0.08 L/min (range − 1.7–0.5 L/min).

Desaturation of peripheral oxygen saturation (SpO_2_) below 85% occurred during 63 mobilisations (19.0%), a drop in mean arterial blood pressure below 50 mmHg during 25 mobilisations (7.5%), and tachycardia (heart rate > 140/min) during 19 mobilisations (5.7%). Bradycardia (heart rate < 40/min) and ventricular arrhythmia were not noted.

All of the described clinical changes during mobilisation were brief and either self-limiting or successfully managed by the ECLS team. They did not require discontinuation of mobilisation and were therefore considered minor complications. Median alterations in heart rate, mean arterial pressure, arterial O_2_ saturation, and ECLS blood flow over the course of each mobilisation (IMS ≥ 3) are listed in Additional file 2.

#### Bleeding from cannulation site

Major bleeding from cannulation site requiring transfusion, surgery and/or discontinuation of mobilisation occurred in 3 out of 43 actively mobilised patients (6.9%) as opposed to 11 out of 72 non-mobilised patients requiring transfusion and/or surgery (15.3%). The three major bleeding complications in actively mobilised (IMS ≥ 3) patients all concerned bleeding from a femoral cannulation site and occurred in two patients without invasive mechanical ventilation and one patient on invasive mechanical ventilation. The types of ECLS used were va-ECMO, vv-ECMO and av-ECCO_2_R. Minor bleeding from cannulation site managed non-surgically and not requiring transfusion or discontinuation of mobilisation occurred in 9 out of 43 actively mobilised patients (20.9%) and in 1 out of 72 non-mobilised patients (1.4%).

#### Cannula displacement

During one mobilisation episode out of 332 active mobilisations (0.3%) an accidental displacement of the venous femoral cannula in a patient on va-ECMO occurred (2.3% of all actively mobilised ECLS patients). Subsequent rapid and effective recannulation with reestablishment of the va-ECMO circuit was achieved without sequelae. The patient was discharged later to a rehabilitation centre.

### Treatment duration and survival

The median length of all ECLS treatments was 7 days (IQR 3–12), treatment durations for respiratory ECLS (vv-ECMO and vv-/av-ECCO2R), non-eCPR circulatory ECLS (va-ECMO and RVAD) and eCPR (va-ECMO) were 9.0 (IQR 5–15), 6.5 (IQR 2–12), and 3.5 (IQR 1–7) days, respectively. More details on ECLS treatment durations are presented in Additional file 3.

The median length of stay in ICU and hospital for all ECLS treatments were 16 days (IQR 7–37) and 33 days (IQR 10–72), respectively.

The ICU-mortality rate for all ECLS patients was 59.1 and 41.6% for non-intubated patients on ECLS (“awake ECLS”). For respiratory ECLS, non-eCPR circulatory ECLS and eCPR the ICU-mortality rates were 57.4, 54.9, and 76.5%, respectively.

## Discussion

In our prospective observational study, active mobilisation (IMS ≥ 3) of critically ill patients on ECLS was undertaken in 37% of patients and found to be feasible and safe if performed by an experienced ECLS team that routinely provides mobilisation treatment and is well equipped to manage major complications. Of note, mobilised ECLS patients predominantly had femoral ECLS cannulation, concurrent renal replacement therapy and did not have additional invasive mechanical ventilation. During active mobilisation, changes in extracorporeal circuit function and cardiopulmonary variations were limited and did not lead to discontinuation of active mobilisation. Four major complications—three major bleedings and one cannula displacement—occurred in a total of 332 active mobilisations in 43 patients actively mobilised. All four adverse events were successfully managed without further sequelae.

In daily practice, clinicians are often concerned about ECLS malfunction, cannula displacement and bleeding from cannula insertion sites during active mobilisation of patients on ECLS [11, 12]. To our knowledge, this is the largest prospective observational study investigating the feasibility, safety, and resource utilisation of active mobilisation of ECLS patients. Several retrospective observational studies and one small pilot randomised controlled study on active mobilisation on ECLS have been published elsewhere [13, 14]. Abrams et al. retrospectively studied the feasibility and safety of active mobilisation of 35 patients on veno-venous and veno-arterial ECMO and found no relevant complications associated with mobilisation [16]. Wells et al. studied 167 patients with active mobilisation on veno-arterial ECMO and also found no major complications with a minor event rate of < 0.5% per mobilisation [17]. Bonizzoli et al. retrospectively studied 101 patients on veno-venous ECMO, of whom 40 patients (39%) were mobilised with an ICU Mobility Scale (IMS) ≥ 3 (sitting over edge of bed) [18]. More than 90% of these patients had a double lumen cannula and no complications were recorded. Munshi et al. evaluated 61 patients with severe ARDS requiring veno-venous ECMO [19]. The ICU physiotherapy team mobilised 82% (50) of these patients. A maximum activity level of ≥ 2 (active exercises in bed) was achieved in 39% [18] of these patients and 17% [8] achieved a maximum activity level ≥ 4 (actively sitting over the side of the bed) without relevant complications. Univariate analysis revealed severity of illness factors differentiating higher intensity and lower intensity physiotherapy. Gottschalk et al. retrospectively studied 37 actively mobilised patients on veno-arterial ECMO and observed no severe adverse events, such as cannula dislocation, bleeding, or cardiorespiratory deterioration [20]. In a recently published small pilot randomised controlled study 4 out of 10 ECMO patients in the early mobilisation group were actively mobilised with an activity level of IMS > 3 on a total of 7 occasions [14]. No serious adverse events and a signal for improved functional independence in the activities of daily living at hospital discharge were reported.

It is encouraging that no major complications were associated with active mobilisation of ECLS patients in the aforementioned studies, which supports further implementation of active mobilisation in this specific group of critically ill adult patients. However, the design of the retrospective observational studies and the small sample size with limited number of active mobilisations in the randomised study may have underestimated adverse clinical events, such as bleeding complications. As opposed to the above described findings, our prospective study is the first study to report major bleeding complications associated with active mobilisation in a larger cohort. Interestingly, in non-mobilised patients the rate of major bleeding from the cannulation site was higher than in mobilised patients. However, groups were not balanced according to coagulation state and severity of illness. Our case of cannula displacement during active mobilisation demonstrates that this potentially life-threatening complication can occur, and that great caution is necessary during active mobilisation with respect to securing ECLS cannulas. It shows that the ECLS teams’ competency for immediate and efficient complication management is crucial for patient safety and survival. As a consequence, drawn from this incident, the training of the ECLS team regarding strict adherence to the local standardised screening protocol before mobilising ECLS patients including accurate cannula securement and safety management has been further intensified.

Active mobilisation was resource intense in terms of personnel and time, demonstrating the need for sufficient staffing of an ECLS team. Our observational data are in line with the results of an international survey by Marhong et al. reporting that most ECMO centres require 3–5 team members for active mobilisation [11]. In addition, and in line with the results of our study population comprising 37% actively mobilised ECLS patients (IMS ≥ 3), the rate of mobilisation reported in our study is in line with the aforementioned studies. Specifically, Abrams et al. reported a 26% mobilisation rate while Wells et al. 28%, Bonizzoli et al. 37%, and Munshi et al. 39% [16–19]. In the international survey of Marhong et al. physical therapy in patients on ECLS was reported by 84% respondents, with 41% initiating it within 72 h after cannulation and mobilisation goals varied from range of motion exercises (81%) to ambulation (22%) [11].

In our study, patients actively mobilised had significantly lower severity of illness scores on ICU admission, lower pre-ECLS lactate levels and catecholamine support, and lower ICU-mortality. This may be explained by the likely perception within the ECLS team that sicker patients are deemed less appropriate for active mobilisation. Considering our study design and research question, and the expected baseline treatment indication bias (e.g. confounding), a possible association between mobilisation and mortality was thus not studied and cannot be inferred. To validly assess the efficacy of mobilisation on survival and also on the important clinical outcome of long-term functional status, randomised controlled studies are warranted.

Further, potential changes in sedation and mobilisation practices in ECLS patients over recent years cannot be excluded. Even though in our institution protocols for cannulation, anticoagulation, sedation and mobilisation were not substantially altered within the last years, it is difficult to assess potential subtle changes of practice in our routine clinical care over time with respect to sedation and mobilisation, as subjective clinical judgement plays an important role for such interventions. To assess global contemporary practice, further surveys and observational studies evaluating these important aspects of current routine clinical care of ECLS patients in other centres are warranted.

Of note, active mobilisation (IMS ≥ 3) was not performed in 63% of the ICU patients. The main reason provided by our ICU team was sedation. Therefore, as expected, most mobilisations were performed in awake patients without concurrent invasive mechanical ventilation. Assumptions on whether the individual daily level of sedation was related to the severity of illness or possible over sedation cannot be made due to the design of the study. However, this result highlights the importance of targeting light or no sedation where clinically feasible and safe to facilitate active mobilisation. As opposed to the international survey of Marhong et al. on barriers to mobilising ECMO patients, haemodynamic and respiratory instability, the level of dependence on ECLS, femoral cannulation, shortage of staff or fear of unintentional decannulation were not frequent reasons for non-mobilisation [11].

Finally, the initial selection of patients into our observational study, together with the fact that our report is based on a single centre with specific expertise in the care of ECLS patients may limit the external validity and generalisability of our findings to a broader context.

## Conclusions

In conclusion, our study shows that active mobilisation of critically ill patients on ECLS with predominantly femoral cannulation is feasible and safe if performed by an experienced ECLS team that routinely provides mobilisation treatment and is well equipped to manage major complications. However, randomised controlled multicentre studies are needed to evaluate the effect of early goal directed sedation and mobilisation bundles in this specific patient population and to validly assess its efficacy on survival and long-term functional outcomes.

## Supplementary information

**Additional file 1: Table 1.** Respiratory, haemodynamic, and renal variables within 6 hours before initiation of ECLS according to level of mobilisation.

**Additional file 2: Table 2.** Variation of haemodynamic state, oxygenation, and ECLS blood flow over the course of each active mobilisation unit IMS ≥ 3.

**Additional file 3: Table 3.** Duration of ECLS treatments according to type of ECLS and ICU-mortality.

## Data Availability

The datasets used and/or analysed during the current study are available from the corresponding author on reasonable request.

## Abbreviations

AHF: Acute heart failure
APACHE: Acute Physiology and Chronic Health Evaluation
ARDS: Acute respiratory distress syndrome
av-ECCO_2_R: Arterio-venous extracorporeal carbon dioxide removal
BMI: Body mass index
COPD: Chronic obstructive pulmonary disease
CS: Cardiac surgery
DL: Double lumen
ECLS: Extracorporeal life support
ECMO: Extracorporeal membrane oxygenation
eCPR: Extracorporeal cardiopulmonary resuscitation
ICU: Intensive care unit
ILD: Interstitial lung disease
IMS: ICU mobilisation scale
IQR: Interquartile range
PAH: Pulmonary arterial hypertension
RASS: Richmond Agitation Sedation Scale
RVAD: Right ventricular assist device
SAPS: Simplified Acute Physiology Score
SOFA: Sepsis-related Organ Failure Assessment
va-ECMO: Veno-arterial extracorporeal membrane oxygenation
vv-ECCO_2_R: Veno-venous extracorporeal carbon dioxide removal
vv-ECMO: Veno-venous extracorporeal membrane oxygenation
