# Supplementary material for: Feasibility, safety, and resource utilisation of active mobilisation of patients on extracorporeal life support: a prospective observational study
Source: Ann Intensive Care. 2020 Dec 1;10:161. doi: 10.1186/s13613-020-00776-3 (PMC7708587; doi:10.1186/s13613-020-00776-3)
Supplement: Supplementary file 1 — Additional file 1: Table 1. Respiratory, haemodynamic, and renal variables within 6 hours before initiation of ECLS according to level of mobilisation. [file 13613_2020_776_MOESM1_ESM.pdf]

Table 3: Duration of ECLS treatments according to type of ECLS and ICU-mortality

**Primary type of ECLS**	**Death in ICU**	**Discharge from ICU**	**All** n=115	**p-value**
**Days on ECLS**				
Respiratory ECLS	10.0 (5-19)	9.0 (6-11)	9 (5-15)	0.70
vv-ECMO	7.5 (2-14)	8.0 (6-11)	7.5 (4-11)	0.80
vv-$ECCO_2R$	14.5 (8-23)	8.5 (3-12)	9.5 (4-21)	0.39
av-$ECCO_2R$	17.0 (10-20)	9.0 (4-17)	13 (5-23)	0.31
Non-eCPR circulatory ECLS	4.0 (1-9)	10.0 (6-14)	6.5 (2-12)	0.006
va-ECMO (non-eCPR)	4.0 (1-9)	10 (6-16)	6.0 (2-10)	0.001
RVAD	20.0 (14-26)	7.5 (3-12)	13.0 (5-23)	0.30
eCPR (va-ECMO)	2 (1-5)	7.0 (4-9)	3.5 (1-7)	0.10
All ECLS	5.0 (1-12)	9.0 (6-12)	7 (3-12)	0.016

Data is presented as median (25th and 75th percentile) for continuous variables. ECLS = Extracorporeal Life Support; vv-ECMO = veno-venous Extracorporeal Membrane Oxygenation; vv-$ECCO_2R$ = veno-venous Extracorporeal Carbon Dioxide Removal; av-$ECCO_2R$ = arterio-venous Extracorporeal Carbon Dioxide Removal; va-ECMO = veno-arterial Extracorporeal Membrane Oxygenation; eCPR = extracorporeal Cardiopulmonary Resuscitation; RVAD = Right Ventricular Assist Device.
