# Supplementary material for: Feasibility, safety, and resource utilisation of active mobilisation of patients on extracorporeal life support: a prospective observational study
Source: Ann Intensive Care. 2020 Dec 1;10:161. doi: 10.1186/s13613-020-00776-3 (PMC7708587; doi:10.1186/s13613-020-00776-3)
Supplement: Supplementary file 2 — Additional file 2: Table 2. Variation of haemodynamic state, oxygenation, and ECLS blood flow over the course of each active mobilisation unit IMS ≥ 3. [file 13613_2020_776_MOESM2_ESM.pdf]

Table 1: Respiratory, haemodynamic, and renal variables within 6 hours before initiation of ECLS according to level of mobilisation.

**Variable**	**Mob** n=43 (37.4%)	**Non-Mob** n=72 (62.6%)	**All patients** n=115	**p-value**
**Respiratory, haemodynamic, and renal variables before initiation of ECLS** – median (IQR) or n (%)				
Pre-PaO_2/FiO_2 (mmHg)	146 (98-242)	136.5 (78-204)	142 (80-235)	0.33
Pre-$PaCO_2$ in respiratory ECLS	58.4 (38.7-82)	55.4 (36.6-72.5)	55.4 (37-80)	0.63
Pre-pH in respiratory ECLS	7.3 (7.2-7.4)	7.3 (7.1-7.3)	7.3 (7.1-7.4)	0.09
Lactate max <6h pre-ECLS (mmol/l)	1.3 (0.9-2.0)	4.7 (1.5-9.4)	2.0 (1.2-6.2)	< 0.001
Catecholamines in 24h pre-ECLS n	22 (28.9)	54 (71.1)	76 (66.1)	< 0.001
Adrenalin max <6h pre-ECLS	0.1 (0.008-0.5)	0.4 (0.01-9.3)	0.2 (0.1-1.4)	0.31
Noradrenalin max <6h pre-ECLS	0.5 (0.2-0.6)	0.7 (0.3-2.0)	0.5 (0.2-1.4)	0.06
Dobutamine max <6h pre-ECLS	4 (3.6-4.8)	5.2(3.9-8.2)	4.2(3.7-7.5)	0.22
On RRT Pre-ECLS n (%)	4 (57.1)	3 (42.9)	7 (6.1)	0.42
Without IMV Pre-ECLS n (%)	9 (20.9)	3 (4.2)	12 (10.4)	0.09
Mean days on IMV Pre-ECLS	3.7 (0-59)	1.7 (0-34)	2.5 (0-59)	0.29

Data is presented as n (%) for categorical variables and median (25th and 75th percentile) for continuous variables. Mob = at least one active mobilisation episode with IMS ≥ 3; Non-Mob = never mobilised with IMS ≥ 3; eCPR = extracorporeal Cardiopulmonary Resuscitation; ECLS = Extracorporeal Life Support; va-ECMO = veno-arterial Extracorporeal Membrane Oxygenation; vv-ECMO = veno-venous Extracorporeal Membrane Oxygenation; vv-$ECCO_2R$ = veno-venous Extracorporeal Carbon Dioxide Removal; av-$ECCO_2R$ = arterio-venous Extracorporeal Carbon Dioxide Removal; RVAD = Right Ventricular Assist Device; DL = Double Lumen; IQR = Interquartile Range; PaO_2 = Partial Arterial Oxygen Pressure; FiO_2 = Fraction Inspired Oxygen; $PaCO_2$ = Partial Arterial Carbon Dioxide Pressure; RRT = Renal Replacement Therapy; IMV = Invasive Mechanical Ventilation.
