# Supplementary material for: Feasibility, safety, and resource utilisation of active mobilisation of patients on extracorporeal life support: a prospective observational study
Source: Ann Intensive Care. 2020 Dec 1;10:161. doi: 10.1186/s13613-020-00776-3 (PMC7708587; doi:10.1186/s13613-020-00776-3)
Supplement: Supplementary file 3 — Additional file 3: Table 3. Duration of ECLS treatments according to type of ECLS and ICU-mortality. [file 13613_2020_776_MOESM3_ESM.pdf]

Table 2: Variation of haemodynamic state, oxygenation, and ECLS blood flow over the course of each active mobilisation unit IMS ≥ 3.

Variable	Pre-Mob	During Mob	Post-Mob	p-value
Heart rate (/min)	94 (84-108)	107 (92-120)	94 (83-108)	< 0.001
Mean arterial pressure (mmHg)	81 (70-91)	71 (60-83)	80 (69-91)	< 0.001
Arterial O_2 saturation (%)	96 (93-98)	91 (86-95)	97 (94-99)	< 0.001
BF high-flow ECLS (vv-/va-ECMO, RVAD)	3.9 (3.2-4.7)	3.7 (3.1-4.5)	3.8 (3.1-4.7)	< 0.001
BF mid-flow ECLS (vv-/av-$ECCO_2R$)	1.4 (1.3-1.6)	1.4 (1.2-1.6)	1.4 (1.2-1.5)	0.03

Data is presented as median (25th and 75th percentile) for continuous variables. Mob = at least one active mobilisation episode with IMS ≥ 3; BF = Blood Flow in the extracorporeal circuit; ECLS = Extracorporeal Life Support; va-ECMO = veno-arterial Extracorporeal Membrane Oxygenation; vv-ECMO = veno-venous Extracorporeal Membrane Oxygenation; vv-$ECCO_2R$ = veno-venous Extracorporeal Carbon Dioxide Removal; av-$ECCO_2R$ = arterio-venous Extracorporeal Carbon Dioxide Removal; RVAD = Right Ventricular Assist Device.
